# Refusal of beds and triage of patients admitted to intensive care units in Brazil: a cross-sectional national survey

**DOI:** 10.5935/0103-507X.20220264-en

**Published:** 2022

**Authors:** Rafaela de Lemos Lepre, Ana Luiza Mezzaroba, Lucienne Tibery Queiroz Cardoso, Tiemi Matsuo, Cíntia Magalhães Carvalho Grion

**Affiliations:** 1 Department of Surgical Clinic, Universidade Estadual de Londrina - Londrina (PR), Brazil.; 2 Department of Internal Medicine, Universidade Estadual de Londrina - Londrina (PR), Brazil.; 3 Department of Statistics, Universidade Estadual de Londrina - Londrina (PR), Brazil.

**Keywords:** Bed occupancy, Critical care, Triage, Surveys and questionnaires, Intensive care units

## Abstract

**Objective:**

To obtain data on bed refusal in intensive care units in Brazil and to evaluate the use of triage systems by professionals.

**Methods:**

A cross-sectional survey. Using the Delphi methodology, a questionnaire was created contemplating the objectives of the study. Physicians and nurses enrolled in the research network of the *Associação de Medicina Intensiva Brasileira* (AMIBnet) were invited to participate. A web platform (SurveyMonkey^®^) was used to distribute the questionnaire. The variables in this study were measured in categories and expressed as proportions. The chi-square test or Fisher’s exact test was used to verify associations. The significance level was set at 5%.

**Results:**

In total, 231 professionals answered the questionnaire, representing all regions of the country. The national intensive care units had an occupancy rate of more than 90% always or frequently for 90.8% of the participants. Among the participants, 84.4% had already refused admitting patients to the intensive care unit due to the capacity of the unit. Half of the Brazilian institutions (49.7%) did not have triage protocols for admission to intensive beds.

**Conclusions:**

Bed refusal due to high occupancy rates is common in Brazilian intensive care units. Even so, half of the services in Brazil do not adopt protocols for triage of beds.

## INTRODUCTION

With the advancement of medical sciences, the emergence of more complex procedures and the increase in life expectancy, there is naturally a greater demand for health services. The growing need for intensive care beds fits into this scenario, and such demand often exceeds supply. The cost is impressive, particularly after the outbreak of coronavirus disease 2019 (COVID-19). In Brazil, it is estimated that a patient in an intensive care unit (ICU) bed costs approximately R$2,000.00 (approximately US$500.00) per day.^(1)^

In intensive care, when the admission rate of patients falls, the outcomes are worse for them.^(2)^ It is known that ICU admission refusal is associated with higher death rates^(3-5)^ and that, more specifically, for critically ill patients, there is a 1.5% increase in the risk of death for each hour of delay in ICU admission.^(6)^ Due to these factors, refusing and screening ICU beds are invariably complex decisions that must take into account several aspects, from clinical to ethical to meeting the wishes of patients and family members.

Aiming to help with this difficult decision-making process, several specialized medical societies have developed guidelines that aid in triage. The North American Society of Critical Care Medicine (SCCM)^(7)^ and the task force of the World Federation of Societies of Intensive and Critical Care Medicine (WFSICCM)^(8)^ updated their guidelines in 2016. In the same year, the Brazilian Federal Council of Medicine (CFM - *Conselho Federal de Medicina*) published its resolution 2156/2016^(9)^ regulating the admission priorities of patients to ICU beds. The guidelines of the specialized societies and CFM Brazil are similar in many aspects, and all recommend that the intensive care services of each institution develop their own protocols, based both on the specialized recommendations and on the individuality of each service. Even so, in the daily routine of ICUs, refusal and triage decisions are regularly based not on scientific evidence but on clinical experience.^(10)^

In the literature, data regarding triage and refusal of ICU beds in Brazil are scarce. The reality of Brazilian intensive care Medicine in these aspects is uncertain, as few studies have been published with this objective.^(6,10)^ It is not known whether Brazilian ICUs have their own protocols or if they follow any of the published guidelines. In addition, Brazilian data is scarce regarding the occupancy rate of intensive care units, frequency of bed refusal, training of professionals in triage or whether there are differences between public, private and mixed services. There is also no clear knowledge about which professional should be responsible for refusal and triage, how long critical patients usually wait for intensive beds in other sectors of the hospital, or whether regional differences exist.

In view of this, the objective of this study is to obtain data on the refusal of beds in ICUs in Brazil, as well as to evaluate the use of triage systems by professionals.

## METHODS

A cross-sectional survey was conducted with a questionnaire. This type of evaluation is increasingly used as a tool to access data in various areas, including health care, as well as to translate scientific research into clinical practice.^(11)^

The questions were selected using the Delphi method. A list of questions of interest to the study was developed and sent to five specialists in the field of intensive care medicine (certified intensivists) for consideration and suggestions for changes. After each evaluation, the suggestions were incorporated into the questionnaire and sent for a new round of evaluation. The rounds ended when a consensus of at least 80% approval was reached for each question.^(12)^

The final questionnaire consisted of 58 questions. Questions 1 to 14 referred to the profile of the interviewees; 15 to 36 to the profile of the institution (hospital/ICU); and 37 to 58 to the refusal and triage of ICU beds (Supplementary material). The information on the profile of the interviewees was self-declarations, and no definition of the questions was offered. The variables were categorized according to the suggestions of the experts in the Delphi methodology who helped prepare the questionnaire.

A web platform (SurveyMonkey^®^) was used to distribute the questionnaire.^(13)^ The research was conducted by invitation to physicians and nurses working in intensive care units and emergency departments enrolled in the virtual network of the *Associação de Medicina Intensiva Brasileira* (AMIBnet) via its own network platform. The questionnaire was made available from February to August 2021, and potential participants were sent two reminders during this period.

When accessing the link, the interviewee was initially presented with the Free and Informed Consent Form for participation in the study. The second page provided guidelines for completing the questionnaire, stating that there would be 58 questions answered in approximately 8 minutes. If working in more than one ICU, the participant was asked to respond to questions based on experiences in the ICU where he or she most often worked. On the third page, the questions began. Participants responded to each question until the questionnaire was completed.

All the variables in this study were measured in categories and expressed as proportions. The chi-square test or Fisher’s exact test (when more than 20% of the cells had an expected frequency lower than 5%) was used to verify the association between these variables. A multinomial logistic regression analysis was performed to investigate possible factors associated with the daily frequency of non-admission to the ICU. The significance level used was 5%. The analyses were performed using the IBM Statistical Package for the Social Sciences software (SPSS; IBM Corp. Armonk, NY), version 19.

The research project was presented to the Research Ethics Committee of the *Universidade Estadual de Londrina*, registered under number 23246919.9.0000.5231 and approved by opinion 3,698,448, published on November 11, 2019.

## RESULTS

At the end of the study, 231 physicians and nurses working in the field of intensive care completed the questionnaire. Among them, 87.4% self-reported as intensivist physicians or nurses. The majority worked exclusively in ICUs (74.0%), in more than one unit (55.8%) and for more than 10 years in this sector (62.8%). Eighty-eight of the 231 (38.3%) were technical coordinators, and 109 were the day care intensivist (47.4%) (Table 1).

**Table 1:** Demographic, educational and professional characteristics of the participants

Variable	
Age group (years)	
< 30	11 (4.8)
30 - 50	167 (72.6)
> 50	52 (22.6)
Training	
Graduation	8 (3.5)
Specialization	142 (61.7)
Master’s	47 (20.4)
Doctorate	33 (14.4)
Specialist in intensive care	
Yes	202 (87.4)
No	29 (12.6)
Works only in ICU	
Yes	171 (74.0)
No	60 (26.0)
How many hours per week do you work in the ICU	
Up to 12	16 (6.9)
12 - 36	58 (25.1)
> 36	157 (68.0)
How long have you been working in the ICU (years)	
< 5	22 (9.5)
5 - 10	64 (27.7)
> 10	145 (62.8)
Works in more than one ICU	
Yes	129 (55.8)
No	102 (44.2)
Is a professor in the ICU	
Yes	105 (45.5)
No	126 (54.5)
Has an employment relationship in the ICU	
Yes	156 (67.8)
No	74 (32.2)
Is the ICU technical coordinator	
Yes	88 (38.3)
No	142 (61.7)
Is an ICU day care intensivist	
Yes	109 (47.4)
No	75 (32.6)
Not applicable	46 (20.0)

ICU - intensive care unit. The results are expressed as n (%).

Regarding the profile of the institution, 50.6% were public, 22.6% were private, and 26.8% were mixed. The largest number was located in the Southeast (87; 37.7%), while 14 (6.1%) were in the North. Of the total, 66.7% were ICUs in cities with more than 500,000 inhabitants, 79.7% of the hospitals had more than 50 general beds, and 62.8% were university hospitals. Most of these institutions did not have semi-intensive care services (179; 77.5%) or a Rapid Response Team (135; 58.4%), but the majority had organ donation teams (143; 62.4%). Most ICUs had established clinical protocols (194; 84.3%) (Table 2).

**Table 2:** Profile of the institutions participating in the study

Variable	
Public/private nature	
Private	117 (50.6)
Private	52 (22.6)
Mixed	62 (26.8)
Region	
North	14 (6.1)
Northeast	35 (15.2)
Midwest	19 (8.2)
Southeast	87 (37.7)
South	76 (32.8)
How many ICUs are there in the service?	
1	31 (13.5)
2	48 (20.8)
3 or more	151 (65.7)
How many beds are there in the ICU?	
Up to 5	3 (1.3)
5 - 10	91 (39.6)
> 10	136 (59.1)
Are there closed beds in the ICU?	
Yes	49 (21.2)
No	182 (78.8)
Is there a semi-intensive care service?	
≤ 5 beds	15 (6.5)
6 - 10 beds	14 (6.0)
> 10 beds	23 (10.0)
No	179 (77.5)
How many general beds are there in the hospital?	
Up to 30	24 (10.3)
30 - 50	23 (10.0)
> 50	184 (79.7)
Is the hospital a reference for referrals?	
Yes	196 (84.8)
No	35 (15.2)
How many inhabitants does the city have?	
Up to 100,000	18 (7.8)
100 - 500,000	59 (25.5)
> 500,000	154 (66.7)
Is the hospital a university hospital?	
Yes	145 (62.8)
No	86 (37.2)
Does the ICU have clinical protocols?	
Yes	194 (84.3)
No	36 (15.7)
What is the main source of patients to the ICU?	
Hospital	175 (75.8)
Other institutions	56 (24.2)
Availability of services 24 hours/day	
Imaging tests	209 (90.9)
Laboratory tests	230 (99.6)
Physiotherapy	126 (54.5)
Reservation of beds for elective surgeries	
Yes	45 (19.6)
No	185 (80.4)
Organ donation team	
Yes	143 (62.4)
No	86 (37.6)
There is a Rapid Response Team in the hospital	
Yes, 24 hours/day	85 (36.8)
Yes, 12 hours/day	11 (4.8)
No	135 (58.4)

ICU - intensive care unit. The results are expressed as n (%).

In these services, the request for a vacancy in the ICU was made by the patient’s attending physician (74; 32.2%) or by a physician in the emergency department (69; 30.0%). The request was usually electronic (107; 46.3%) or verbal (86; 37.2%). In 40.3% of the cases, participants reported that the institution had never promoted general refresher courses or classes, and 71.3% responded that they had never participated in courses or classes on bed triage.

According to the perception of the participants, more than 90% of their beds were always (48.4%) or frequently (42.2%) occupied, and 195 (84.4%) reported having failed to admit patients to the unit due to capacity. This occurred daily for 54 (23.4%) of them. Patients who were waiting for intensive care beds were often in the hospital emergency room (158; 69.3%) under the care of the sector team (117; 50.6%). The waiting time outside the ICU was variable - from less than 6 hours to more than 24 hours - and there was a similar proportion in the responses obtained (Table 3). The units with bed occupancy rates greater than 90% most of the time were public or mixed ICUs. In these institutions, the frequency of non-admission was higher. The frequency of more than one patient waiting for a bed outside the ICU was also higher, as was the waiting time outside the ICU (Table 3).

**Table 3:** Waiting time for admission to intensive care units according to their occupancy rates

Occupancy rate > 90%?	Always	Often	Almost never	Never	p value^*^
Type of ICU					
Exclusive private	70 (59.8)	42 (35.9)	5 (4.3)	0 (0.0)	p < 0.001
Exclusive private	13 (25.0)	30 (57.7)	8 (15.4)	1 (1.9)	
Mixed	29 (46.8)	26 (41.9)	7 (11.3)	0 (0.0)	
Frequency of non-admission					
Daily	43 (79.6)	11 (20.4)	0 (0.0)	0 (0.0)	p < 0.001
1 time/week to 3 times/month	42 (53.8)	34 (43.6)	2 (2.6)	0 (0.0)	
Rarely	27 (29.0)	50 (53.8)	15 (16.1)	1 (1.1)	
Patient waiting time outside the ICU (hours)					
< 6	23 (30.7)	36 (48.0)	15 (20.0)	1 (1.3)	p < 0.001
6 - 12	20 (42.6)	25 (53.2)	2 (4.3)	0 (0.0)	
12 - 24	26 (51.0)	22 (43.1)	3 (5.9)	0 (0.0)	
> 24	43 (74.1)	15 (25.9)	0 (0.0)	0 (0.0)	
Frequency (more than one patient waiting for a vacancy)					p < 0.001
Daily	54 (72.0)	20 (26.7)	1 (1.3)	0 (0.0)	
1 time/week to 3 times/month	37 (50.0)	36 (48.6)	1 (1.4)	0 (0.0)	
Rarely	21 (25.6)	42 (51.2)	18 (22.0)	1 (1.2)	

ICU - intensive care unit.

* Fisher’s exact test p value. The results are expressed as n (%).

When asked if they had received guidance regarding the triage of intensive beds, most participants answered no - both for verbal guidance (60.6%) and for written or e-mail guidance (73.6%) (Table 1S - Supplementary material). Nevertheless, most knew the CFM (71.0%) and SCCM (53.2%) triage guidelines; 37.2% knew the WFSICCM guidelines.

Among the interviewees, 49.8% reported that there was no triage protocol established in the ICU. Among the 78 participants who reported having a protocol in place, 62 (79.4%) considered themselves familiar with this protocol, which was generally based on the CFM guidelines (39.0%) or was a protocol specific to the service (24.1%). Triage was the responsibility of the day care physician or ICU coordinator in 40.6% of cases. When there was no protocol, physicians based their decisions on the severity of the case (28.3%) or prognosis (20.4%). The following options obtained lower numbers of responses: chronological order of request, other factors, patient age, organ donation and underlying pathology.

When comparing data related to the presence of triage protocols with the number of general hospital beds, the number of inhabitants in the city, the fact that the hospital is a reference for other regions, the fact that the hospital is a teaching hospital, the location of the service in the various regions of the country and the public‒private nature of the ICU, no associations were found. There was an association between the presence of triage protocols and clinical protocols established in the ICU (p = 0.004). An association was also observed between the adoption of triage protocols and a higher frequency of more than one patient waiting for an ICU bed (Table 2S - Supplementary material). Specialists in intensive care medicine, certified by the *Associação de Medicina Intensiva Brasileira* (AMIB), technical coordinators or day care intensivists were more familiar with the triage guidelines of specialized societies (Table 3S - Supplementary material).

No associations were found between the profile of the institutions (presence of semi-intensive care unit, Rapid Response Team, availability of complementary exams or physical therapy) and the presence of triage protocols or triage guidelines and the location of the ICUs (North, Northeast, Central-West, Southeast and South).

In the public or mixed ICUs, failure to admit a patient due to unit capacity was more frequent, and the frequency of non-admission was also higher. In these institutions, the wait time of critically ill patients outside the ICU was also usually longer and was more common when more than one patient was waiting for a place in the intensive care unit (Table 4).

**Table 4:** Admission and triage in intensive care units according to their types of administration

	Public	Private	Mixed	p value^*^
Failed to admit due to overcrowding				
Yes	103 (88.0)	34 (65.4)	58 (93.5)	p < 0.001
No	14 (12.0)	18 (34.6)	4 (6.5)	
Frequency of non-admission				
Daily	38 (33.3)	3 (6.1)	13 (21.0)	p < 0.001
1 time/week to 3 times/month	42 (36.8)	12 (24.5)	24 (38.7)	
Rarely	34 (29.8)	34 (69.4)	25 (40.3)	
Waiting time of the patient outside the ICU (hours)				
< 6	26 (22.2)	32 (61.5)	17 (27.4)	p < 0.001
6 - 12	16 (13.7)	13 (25.0)	18 (29.0)	
12 - 24	31 (26.5)	6 (11.5)	14 (22.6)	
> 24	44 (37.6)	1 (1.9)	13 (21.0)	
Frequency - more than one patient waiting for a vacancy				
Daily	52 (44.4)	5 (9.6)	18 (29.0)	p < 0.001
1 time/week to 3 times/month	39 (33.3)	13 (25.0)	22 (35.5)	
Rarely	26 (22.2)	34 (65.4)	22 (35.5)	

ICU - intensive care unit.

* p value of the chi-square test. The results are expressed as n (%).

In the multivariate analysis, the independent variables associated with the daily frequency of non-admission were the type of ICU (public, private or mixed), the fact that the hospital was a university hospital and a referral center (Table 5).

**Table 5:** Multinomial logistic regression analysis for factors associated with the frequency of non-admission due to lack of beds

	Frequency of non-admission due to lack of ICU beds			
	Daily	1 time/week to 3 times/month	Rarely	p value
Type of ICU				< 0.001
Exclusive private	38 (70.4)	42 (53.8)	34 (36.6)	
Exclusive private	3 (5.6)	12 (15.4)	34 (36.6)	
Mixed	13 (24.1)	24 (30.8)	25 (26.9)	
Has clinical protocols				0.122
Yes	50 (92.6)	62 (79.5)	77 (83.7)	
No	4 (7.4)	16 (20.5)	15 (16.3)	
Semi-intensive care unit				0.211
Yes (≤ 5 beds)	1 (1.9)	6 (7.7)	8 (8.6)	
Yes (6 - 10 beds)	3 (5.6)	2 (2.6)	9 (9.7)	
Yes (> 10 beds)	4 (7.4)	6 (7.7)	11 (11.8)	
No	46 (85.2)	64 (82.1)	65 (69.9)	
Closed beds				0.387
Yes	10 (18.5)	14 (17.9)	24 (25.8)	
No	44 (81.5)	64 (82.1)	69 (74.2)	
University hospital				< 0.001
Yes	46 (85.2)	54 (69.2)	44 (47.3)	
No	8 (14.8)	24 (30.8)	49 (52.7)	
Reservation of beds for elective surgeries				0.443
Yes	13 (24.1)	12 (15.4)	19 (20.7)	
No	41 (75.9)	66 (84.6)	73 (79.3)	
Is the hospital a reference for referrals?				0.003
Yes	52 (96.3)	70 (89.7)	72 (77.4)	
No	2 (3.7)	8 (10.3)	21 (22.6)	

ICU - intensive care unit. The results are expressed as n (%).

## DISCUSSION

The frequency of bed refusal due to the capacity of intensive care units is high in Brazilian hospitals, especially in public institutions, university hospitals and reference units for referrals. In agreement with this finding, the wait times of critically ill patients outside the ICU is also long. Approximately half of the Brazilian institutions participating in this study do not have triage protocols for intensive care admissions. The findings of this study may have been influenced by the change in the health care structure that occurred during the COVID-19 pandemic. Although the questionnaire was administered 1 year after the onset of the pandemic, the incidence of cases in Brazil was still high, and this may have affected the results.^(14)^

The presence of protocols is of crucial importance, as health professionals have low accuracy in predicting outcomes for critically ill patients, especially in acute clinical worsening and ICU request.^(15)^ Based on the results of the present study, it was possible to observe an association between the number of patients waiting for a vacancy and the adoption of triage protocols, possibly because the pressure of increased demand led to the need for better organization of the unit.

Among the units that have protocols, most are based on the CFM, and some have developed their own protocols. Authors suggest the standardization of triage with local protocols^(16)^ based on the CFM, SCCM and WFSICCM.^(7-9)^ However, even when a hospital has an established protocol, it is not always applied in clinical practice.^(17)^ In the Netherlands, the results of an online questionnaire from 2016 showed that, even though they were familiar with it, only 47% of respondents reported that the established protocol was sufficient for decision-making.^(17)^ Bed triage is one of the most stressful aspects of ICU work.^(18)^

Several studies have shown that the availability of ICU beds affects the admission decision and triage of patients. In the United States, a 2014 study conducted in New York and a 2018 study in New Orleans agree in this regard.^(19,20)^ In Tunisia, a 2018 publication showed that refusal due to lack of beds is a common occurrence.^(21)^ In Morocco, another country with an economy similar to Brazil, the general refusal rate is 35%, and the lack of beds is the main cause for this refusal.^(22)^ A study in Australia and New Zealand found values between 25 and 30% for refusal.^(23)^ In Hong Kong, one publication reported 38% refusal,^(24)^ whereas in a French multicenter study, this rate was 23%, and within this general percentage, only 6.5% were due to full units.^(25)^ Another European multicenter study found an overall refusal rate of 15%, and among the causes of refusal, 47% were due to lack of beds.^(26)^

In Brazil, Caldeira et al. analyzed 359 patients and observed a 30% refusal. The factors that influenced the decision were age and priority, according to the SCCM criteria.^(27)^ A Brazilian study by Rocco et al. showed a 44% refusal rate, with age, comorbidities and severity as determinants.^(28)^

The ICUs in the present study always had or frequently had high occupancy rates. These data agree with other data from Brazilian studies, such as a 2011 cohort study, in which the overall bed occupancy rate was 97.3%.^(6)^ The World Health Organization (WHO) recommends that this rate be below 80% for intensive care units.^(6)^

The CFM, in Article 2 of its resolution that guides ICU admission, establishes that admission and discharge are the responsibility of the intensivist.^(9)^ Data collected by this questionnaire show that the responsibility for refusing or triaging patients often lies with the daily physician or ICU coordinator, corroborating the specialized guidelines.

More than half of the participants reported never having received guidance regarding the triage of ICU beds where they worked. There are few national or international studies on the training of triage intensive beds in the literature. Training or receiving specific guidance can significantly change the choice of professional at the critical moment of triage. This choice is invariably difficult, and intensivists can carry the weight of this choice for a long time in their professional journey.^(7)^ Ramos et al. describe that having received training regarding triage promotes a higher classification of patient-related factors at the time of decision-making.^(10)^ The current questionnaire revealed that, even without specific training, many professionals reported knowledge of the guidelines regarding triage. The data collected indicate that, in Brazil, specialists in intensive care medicine are more knowledgeable about these guidelines when compared to nonspecialists.

Regarding the wait time outside the ICU, results with similar proportions appeared among all the alternatives proposed, from less than 6 hours to more than 24 hours. Cardoso et al. also found much variability in this aspect, with patients waiting for ICU beds in other sectors of the hospital from 2 hours to 3.5 days.^(6)^

In the present study, patients from public or mixed institutions waited longer for ICU admission than patients from private institutions. It was also evident that the most frequent occurrence is the occupation of more than 90% of the ICU beds and, consequently, refusal because of capacity in public hospitals, university hospitals and services that are references for referrals. It is reasonable to assume that this is associated with the known overload of patients requiring ICU beds in these units. Ramos et al. concluded that factors related to the shortage of beds were considered more relevant by physicians working in public ICUs (number of beds available, number of occupied operating rooms). In the private ICUs, management factors were more relevant for decision-making (pressure from the requesting physician and fear of lawsuits due to malpractice).^(10)^

Most of the institutions where the interviewees in the present study worked did not have semi-intensive care services. A 2010 multicenter study showed that the availability of intermediate units improves the prognosis of critically ill patients.^(26)^ In the same vein, both the CFM and SCCM guidelines, as well as those of the WFSICCM, warn about the importance of the semi-intensive care unit for the proper management of severe cases.^(7-9)^ In addition, Rapid Response Teams play an important role in the evaluation and triage of patients waiting for intensive care units. In the presence of these teams, patients with higher severity scores and more comorbidities are more often admitted to the ICU.^(29)^

According to the guidelines of specialized societies, the possibility of organ donation is a factor that can change the priority of ICU admission.^(7-9)^ Even so, this factor is a conflicting point in medical practice. A study showed that professionals tend to admit a patient with little chance of survival more often than a potential organ donor.^(30)^

In the literature, 88% of the institutions have a triage protocol in place, but only 25% make regular use of the guidelines.^(31)^ In the present study, there was an association between the presence of protocols for triage of critical beds and the presence of established clinical protocols. This finding demonstrates that the policy of establishing protocols is an important point in these services, both in the general clinic and in the intensive care environment. The protocol-based work policy improves patient outcomes.^(7,8)^

The strength of this study derives from its wide coverage of the national territory in a highly relevant topic regarding the refusal and triage of ICU beds, considering the shortage of beds in most of the country. The limitation of the *survey* design with the application of a structured questionnaire is the restriction of access to data from the ICUs evaluated by the study. The information collected on structural and institutional issues was based on the perceptions of the research participants, not on direct observations. It is also possible that more than one participant responded about the same institution, leading to a greater representation of this institution in the survey results. Another limitation of the study was the fact that the sample was nonrandom and, therefore, may not be representative of the entire country. There may have been a recall bias in the responses to the questionnaire, a limitation inherent to the type of design. However, such a design can be considered adequate for the initial investigation of the scientific question.

## CONCLUSION

Refusal of a bed in the intensive care unit due to the lack of capacity of the unit is frequent in Brazilian intensive care units. The decision falls, in most cases, on the physicians on duty and the day care worker/coordinator of the intensive care unit. Many services in Brazil do not have an intensive care unit bed triage system. In addition, most physicians working in intensive care units do not receive training regarding triage criteria or methods.
