# Supplementary material for: Refusal of beds and triage of patients admitted to intensive care units in Brazil: a cross-sectional national survey
Source: Rev Bras Ter Intensiva. 2022 Oct-Dec;34(4):484–91. doi: 10.5935/0103-507X.20220264-en (PMC9987007; doi:10.5935/0103-507X.20220264-en)
Supplement: Supplementary file 1 [file rbti-34-04-0484-suppl1.pdf]

Refusal of beds and triage of patients admitted to intensive care units in Brazil: a cross-sectional national survey

Rafaela de Lemos Lepre[1], Ana Luiza Mezzaroba[2], Lucienne Tibery Queiroz Cardoso[2], Tiemi Matsuo[3], Cíntia Magalhães Carvalho Grion[2]

Questionnaire

I. Identification of the interviewee

1. What is your age group?
   ( ) < 30 years.
   ( ) 30 - 50 years.
   ( ) > 50 years.
2. What is your education level?
   ( ) Graduate.
   ( ) Specialization.
   ( ) Master's degree.
   ( ) Doctorate.
3. Are you a specialist in Intensive Care Medicine/Intensive Care Nursing?
   ( ) Yes.
   ( ) No.
4. If a specialist in Intensive Care Medicine, did you graduate from the AMIB?
   ( ) Yes.
   ( ) No.
   ( ) Does not apply.
5. Do you work as a doctor/nurse exclusively in the ICU?
   ( ) Yes.
   ( ) No.
6. How many hours per week do you work in the ICU?
   ( ) Up to 12 hours.
   ( ) 12 - 36 hours.
   ( ) > 36 hours.
7. How many years have you worked in the ICU?
   ( ) < 5 years.
   ( ) 5 - 10 years.
   ( ) > 10 years.
8. Do you work in more than one ICU?
   ( ) Yes.
   ( ) No.
9. Do you work in the ICU as a teacher?
   ( ) Yes.
   ( ) No.
10. Are you employed in the ICU?
   ( ) Yes.
   ( ) No.
11. Are you a technical coordinator of the ICU?
   ( ) Yes.
   ( ) No.

12. If technical coordinator of the ICU, how many ICUs employ you in this position?
    ( ) 1.
    ( ) 2.
    ( ) > 2.
    ( ) Does not apply.
13. Are you a daily/routine ICU physician?
    ( ) Yes.
    ( ) No.
14. If a daily ICU physician, how many ICUs employ you in this position?
    ( ) 1.
    ( ) 2.
    ( ) > 2.
    ( ) Does not apply.

II. Institution profile

15. The ICU where you work is characterized by:
    ( ) Exclusive private.
    ( ) Exclusive public.
    ( ) Public–private mix.
16. The ICU where you work is located:
    ( ) In the North.
    ( ) In the Northeast.
    ( ) In the Midwest.
    ( ) In the Southeast.
    ( ) In the South.
17. How many ICUs are there in your service?
    ( ) 1.
    ( ) 2.
    ( ) 3 or more.
18. How many available beds are there in the ICU where you work?
    ( ) Up to 5.
    ( ) 5 - 10.
    ( ) > 10 beds.
19. Are ICU beds closed/unavailable?
    ( ) Yes.
    ( ) No.
20. Is there a semi-intensive care unit in the service?
    ( ) Yes, with 5 beds or less.
    ( ) Yes, with 6 to 10 beds.
    ( ) Yes, with more than 10 beds.
    ( ) No.
21. How many beds (of general care, not intensive care) are there in the hospital where the ICU is located?
    ( ) Up to 30 beds.
    ( ) 30 - 50 beds.
    ( ) > 50 beds.
22. Does the hospital receive patients referred from other cities in the region?
    ( ) Yes.
    ( ) No.

23. How many inhabitants does the city have?
    ( ) Up to 100,000
    ( ) 100 - 500,000
    ( ) > 500,000
24. Is the hospital a university or affiliated to the educational institution?
    ( ) Yes.
    ( ) No.
25. Does the ICU where you operate most have established clinical protocols?
    ( ) Yes.
    ( ) No.
26. When admission to an ICU bed is indicated, how is the request for a place in the service made?
    ( ) Verbal request.
    ( ) Paper application.
    ( ) Application in electronic system.
    ( ) Others.
27. When admission to an ICU bed is indicated, who is responsible for requesting the vacancy?
    ( ) Senior physician.
    ( ) Nursing.
    ( ) Emergency room physician.
    ( ) Hospitalist/internist physician.
    ( ) Physician of the Rapid Response Team.
    ( ) Administrative technician.
    ( ) Others.
28. Does the institution (hospital or ICU) promote refresher courses for physicians/nurses?
    ( ) Never.
    ( ) Monthly.
    ( ) Annually.
29. What is the main source of patients to the ICU?
    ( ) Hospital.
    ( ) Other institutions.
30. Are imaging tests available 24 hours a day in the ICU?
    ( ) Yes.
    ( ) No.
31. Are laboratory tests available 24 hours a day in the ICU?
    ( ) Yes.
    ( ) No.
32. Are physical therapy services available in the ICU?
    ( ) 1 time a day.
    ( ) 2 times a day.
    ( ) 3 times a day.
    ( ) 18 hours a day.
    ( ) 24 hours a day.
    ( ) There is none.
33. Is there a fixed number of beds reserved for elective surgeries in the ICU?
    ( ) Yes.
    ( ) No.

34. If there is a number of elective surgeries greater than the number of beds available, who defines which surgeries will be a priority?
    ( ) Physician on duty.
    ( ) Daily physician.
    ( ) Clinical direction of the hospital.
    ( ) Hospital bed management.
    ( ) Does not apply.
35. Are there active search teams for potential organ donors and/or protocols for the evaluation of potential organ donors at your service?
    ( ) Yes.
    ( ) No
36. Is there a Rapid Response Team available?
    ( ) Yes, 24 hours a day.
    ( ) Yes, 12 hours a day.
    ( ) No.

III. Refusal and triage of beds

37. The ICU where you work the most has an occupancy rate greater than 90%:
    ( ) Always.
    ( ) Often.
    ( ) Almost never.
    ( ) Never.
38. In this service, the non-admission of a patient with a requested place due to the capacity of the ICU is the responsibility of:
    ( ) The physician on duty.
    ( ) The day care physician/ICU coordinator.
    ( ) The ICU nursing team.
    ( ) Others.
    ( ) I do not know.
39. Have you ever failed to admit a critical patient with a requested ICU vacancy due exclusively to the capacity of the unit?
    ( ) Yes.
    ( ) No.
40. If yes, how often, on average, is non-admission due to lack of beds in the ICU?
    ( ) Daily.
    ( ) 1 time per week.
    ( ) 2 to 3 times a week.
    ( ) 2 to 3 times a month.
    ( ) Rarely.
41. What is the average wait time of patients in regular beds from the request until they are able to enter the intensive care unit at your service?
    ( ) Less than 6 hours.
    ( ) 6 - 12 horas.
    ( ) 12 - 24 horas.
    ( ) More than 24 hours.
    ( ) More than 48 hours.
    ( ) More than 72 hours.

42. While waiting for the ICU vacancy, the patient is usually:
    ( ) In the hospital emergency room.
    ( ) In the semi-intensive unit of the hospital.
    ( ) In the hospital ward.
    ( ) In the operating room of the hospital.
    ( ) In other services/hospitals in the region.
    ( ) Other locations.
43. From the request for the ICU vacancy and while waiting for the availability of the intensive bed, the patient is under the care of:
    ( ) Exclusively the sector team.
    ( ) The team in the sector to the patient's primary physician.
    ( ) The team of the sector with the help of the hospitalist.
    ( ) The team of the sector with the help of the intensivist.
    ( ) The team of the sector with the assistance of the ICU nursing staff.
    ( ) The team of the sector with the help of the intensivist doctor and the ICU nurse.
    ( ) The sector team with the help of the Rapid Response Team.
44. How often do you observe more than one patient waiting for an ICU vacancy to be available at your service?
    ( ) Daily.
    ( ) 1 time per week.
    ( ) 2 to 3 times a week.
    ( ) 2 to 3 times a month.
    ( ) Rarely.
45. Did you receive any verbal guidance regarding the triage of patients waiting for ICU beds?
    ( ) Yes.
    ( ) No.
46. If yes, from whom did you receive the guidance?
    ( ) From the day care physician/ICU coordinator.
    ( ) From the director of the hospital.
    ( ) From the nursing team.
    ( ) Others.
    ( ) Does not apply.
47. Did you receive any written or *e-mail* guidance regarding the triage of patients waiting for an ICU bed?
    ( ) Yes.
    ( ) No.
48. If yes, from whom did you receive the guidance?
    ( ) From the day care physician/ICU coordinator.
    ( ) From the director of the hospital.
    ( ) From the nursing team.
    ( ) Others.
    ( ) Does not apply.
49. Have you ever participated in any class, course or training on the triage of patients awaiting a vacancy in the ICU?
    ( ) Yes.
    ( ) No.

50 If yes, who promoted the class/course/training?
    ( ) The hospital.
    ( ) Specialty Association.
    ( ) Medical Association.
    ( ) Others.
    ( ) Does not apply.

51. In this service, the decision regarding which patients will be admitted, in priority to others when necessary, is the responsibility of:
    ( ) The physician on duty.
    ( ) The day care physician/ICU coordinator.
    ( ) The clinical director of the hospital.
    ( ) Management of hospital/ICU beds.
    ( ) The nursing team.
    ( ) Others.
52. Do you know the guidelines of the Federal Council of Medicine regarding the triage of patients for ICU beds?
    ( ) Yes.
    ( ) No.
53. Are you familiar with the Society of Critical Care Medicine (SCCM) guidelines regarding triage of patients for ICU beds?
    ( ) Yes.
    ( ) No.
54. Are you familiar with the World Federation of Societies of Intensive and Critical Care Medicine (WFSICCM) guidelines regarding triage of patients for ICU beds?
    ( ) Yes.
    ( ) No.
55. In this ICU, where you work the most, is there a protocol in place regarding the triage of patients waiting for an ICU bed?
    ( ) Yes.
    ( ) No.
    ( ) I don't know.
56. If yes, do you consider yourself sufficiently familiarized with the protocol?
    ( ) Yes.
    ( ) No.
    ( ) Does not apply.
57. If yes, on which guidance is your hospital's protocol based?
    ( ) CFM (Federal Council of Medicine).
    ( ) SCCM (Society of Critical Care Medicine).
    ( ) WFSICCM (World Federation of Societies of Intensive and Critical Care Medicine).
    ( ) Protocol of the service.
    ( ) Others.
    ( ) I don't know.
    ( ) Does not apply.
58. If there is no protocol in place, you base your triage decision on:
    ( ) Chronological order of request.
    ( ) Age of the patient.
    ( ) Seriousness of the case.
    ( ) Underlying pathology.
    ( ) Prognosis.
    ( ) Organ donation.
    ( ) Others.
    ( ) Does not apply.

Table 1S - Respondents' knowledge of triage guidelines

Variable	
Received verbal guidance	
Yes	91 (39.4)
No	140 (60.6)
If yes, from whom?	
Day care physician/ICU coordinator	57 (25.1)
Hospital management	17 (7.5)
Nursing team	5 (2.2)
Others	10 (4.6)
Did you receive written/email guidance?	
Yes	61 (26.4)
No	170 (73.6)
If yes, from whom?	
Day care physician/ICU coordinator	39 (16.9)
Hospital management	11 (4.9)
Nursing team	3 (1.4)
Others	8 (3.2)

ICU - intensive care unit. The results are expressed as n (%).

Table 2S - Association between the adoption of clinical triage protocols and admission to the unit

	Clinical triage protocols		
	Yes	No	p value
Frequency of non-admission			0.785
Daily	28 (27.7)	26 (21.0)	
1 time/week	11 (10.9)	17 (13.7)	
2 to 3 times/week	15 (14.9)	22 (17.7)	
2 to 3 times/month	6 (5.9)	7 (5.6)	
Rarely	41 (40.6)	52 (41.9)	
Mean patient waiting time outside the ICU (hours)			0.685
< 6	31 (30.1)	44 (34.4)	
6 - 12	23 (22.3)	24 (18.8)	
12 - 24	19 (18.4)	32 (25.0)	
> 24 - 48	14 (13.6)	15 (11.7)	
> 48 - 72	9 (8.7)	6 (4.7)	
> 72	7 (6.8)	7 (5.5)	
Frequency of more than one patient waiting for a place			0.019
Daily	43 (41.7)	32 (25.0)	
1 time/week	7 (6.8)	14 (10.9)	
2 to 3 times/week	11 (10.7)	27 (21.1)	
2 to 3 times/month	9 (8.7)	6 (4.7)	
Rarely	33 (32.0)	49 (38.3)	

ICU - intensive care unit.

Table 3S - Respondents' knowledge of guidelines from specialized societies

	CFM	SCCM	WFSICCM	p value*
Specialist in ICU				
Yes	150 (74.3)	116 (57.4)	81 (40.1)	0.005
No	14 (48.3)	7 (24.1)	5 (17.2)	
Certified by AMIB				
Yes	111 (84.1)	90 (68.2)	61 (46.2)	0.001
No	38 (55.9)	27 (39.7)	20 (29.4)	
ICU technical coordinator				
Yes	73 (83.0)	59 (67.0)	41 (46.6)	0.002
No	91 (64.1)	63 (44.4)	44 (31.0)	
ICU day care physician				
Yes	91 (83.5)	76 (69.7)	51 (46.8)	0.001
No	52 (69.3)	37 (49.3)	25 (33.3)	

CFM - Federal Council of Medicine; SCCM - Society of Critical Care Medicine; WFSICCM - World Federation of Societies on Intensive and Critical Care Medicine; AMIB - *Associação de Medicina Intensiva Brasileira*. * Value of the chi-square test.
